# Cognitive load and prefrontal neuromodulation shape exploration, risk-taking, and information seeking in experience-based decision making

**DOI:** 10.1371/journal.pone.0360059

**Published:** 2026-10-09

**Authors:** Ankit Singh, Varun Dutt, Akash K. Rao

**Affiliations:** 1 Applied Cognitive Science Laboratory, Indian Institute of Technology Mandi, Mandi, India; 2 Manipal Institute of Technology, Manipal Academy of Higher Education, Manipal, India; Hangzhou Normal University, CHINA

## Abstract

Experience-based decision making requires individuals to balance exploration and exploitation under uncertainty, a balance that may be influenced by cognitive demands and prefrontal neuromodulation. We examined the joint effects of trivia-based cognitive load and anodal transcranial direct current stimulation (tDCS) over the left dorsolateral prefrontal cortex (DLPFC) in 60 healthy adults. The primary 2 × 2 between-subjects design crossed Stimulation (anodal tDCS vs sham) with Cognitive-Load Condition (Trivia-Based Load vs No-Trivia Control). Within the Trivia-Based Load subgroup, Lower-Load and Higher-Load Trivia were presented across trials for a secondary trial-level analysis. Participants completed 50 decisions involving a safe choice, a probabilistic high-reward choice, and a costly information-seeking choice. Primary analyses showed that Trivia-Based Load slowed responses and reduced risky choice. Anodal tDCS increased risky choice, information seeking, and alternation between choice types, but did not significantly affect reaction time. Stimulation × Cognitive-Load Condition interactions were significant for information seeking and alternation, reflecting different load-related patterns under anodal tDCS and sham, whereas interactions were not significant for reaction time or risky choice. Secondary analyses indicated longer decision times following Higher-Load than Lower-Load Trivia, without corresponding trial-level load effects on the choice outcomes. These findings show that cognitive load and anodal DLPFC tDCS have outcome-specific joint effects on experience-based decision making, including both additive and context-dependent patterns.

## 1. Introduction

Human decision making in uncertain environments requires a continuous balance between exploring new alternatives and exploiting familiar options that have yielded favorable outcomes in the past [1–3]. This exploration-exploitation trade-off is especially important in decision making from experience, where outcome probabilities are not described explicitly and must be learned through repeated feedback [4–7]. Such decisions depend on the retrieval of previous outcomes, the updating of value representations, and the allocation of attention to recent feedback [6–8].

Cognitive demands and prefrontal control may both influence this adaptive balance. Concurrent cognitive load can limit working-memory and attentional resources, whereas the dorsolateral prefrontal cortex (DLPFC) supports goal maintenance, feedback integration, and flexible adjustment of behavior [2,9–13]. Transcranial direct current stimulation (tDCS) provides an experimental method for examining whether modulation over the DLPFC is associated with changes in decision behavior, although the direction and magnitude of its behavioral effects depend on stimulation parameters, task demands, and individual differences [14–21].

Previous studies have generally examined cognitive load and prefrontal stimulation separately. Consequently, it remains unclear whether concurrent cognitive demand and anodal DLPFC stimulation produce broadly additive effects or whether the effect of stimulation changes when decision making is performed under additional cognitive demand. The present study addressed this question using a repeated decision-from-experience task containing safe, risky, and information-seeking alternatives.

The primary experiment used a 2 × 2 between-subjects design crossing Stimulation (anodal tDCS vs sham) with Cognitive-Load Condition (Trivia-Based Load vs No-Trivia Control). Within the Trivia-Based Load condition, Lower-Load and Higher-Load Trivia were presented across trials to examine secondary trial-level cognitive-load effects. The study examined reaction time, risky choice, information seeking, and alternation rate as related but distinct indicators of decision behavior under uncertainty.

The objective was to determine whether anodal tDCS over the left DLPFC and trivia-based cognitive demand were associated with additive or context-dependent differences in experience-based decision making. The following Background section develops the theoretical basis for the behavioral measures and hypotheses while avoiding repetition of the general study rationale presented above.

## 2. Background

In experience-based decision making, individuals repeatedly sample outcomes and use feedback to decide whether to continue exploiting a familiar option or explore an uncertain alternative [1,2,4,5]. Within Instance-Based Learning Theory and related reinforcement-learning accounts, choice emerges from the retrieval of previous outcome instances and the updating of option values [3,6–8]. When relevant outcomes are readily retrieved and integrated, individuals can adjust their choices flexibly. When concurrent demands interfere with these processes, retrieval may become noisier and value comparisons less stable, which can slow responding and shift behavior toward safer or simpler alternatives.

The DLPFC is relevant to these processes because it contributes to working memory, attentional allocation, goal maintenance, and the integration of recent feedback [2,9,10,13]. Anodal tDCS over the DLPFC has been associated with changes in working memory, risk-related choice, and exploratory behavior, but these effects are not uniform across tasks or stimulation protocols [15–22]. Therefore, the present study does not treat behavioral changes following stimulation as direct evidence of a single neural mechanism. Instead, it examines whether anodal stimulation over the left DLPFC is associated with systematic differences in risky choice, costly information seeking, and switching behavior.

Within the present task, selection of the probabilistic high-reward alternative was treated as an index of risk-taking because participants accepted the possibility of receiving no reward in exchange for a substantially larger potential gain than the guaranteed safe option. Selection of the costly reveal alternative was treated as an index of information seeking because participants paid one point to obtain otherwise unavailable probability information before making their final choice. Alternation rate represented exploratory switching because it measured changes between choice types across successive trials; however, higher alternation could also reflect weaker commitment or unstable value learning. These measures were therefore interpreted as complementary rather than interchangeable indicators of exploration-exploitation behavior.

Resource-limitation and dual-process accounts predict that concurrent cognitive demand should reduce the resources available for evaluating uncertain alternatives, thereby slowing responses and encouraging more conservative choice [11,12,23–28]. In the present design, this prediction was tested primarily by comparing the Trivia-Based Load condition with the No-Trivia Control condition. The secondary within-participant comparison of Lower-Load and Higher-Load Trivia examined whether greater trial-level processing requirements produced additional changes within the loaded condition.

Based on evidence that DLPFC stimulation can alter risk-related and exploratory behavior, we expected participants receiving anodal tDCS to show greater engagement with uncertain and information-rich alternatives than participants receiving sham stimulation. Thus, we expected:

H1. Participants receiving anodal tDCS over the left DLPFC will exhibit greater risky choice, information seeking, and exploratory switching than participants receiving sham stimulation.

Because concurrent trivia requires additional information processing before each decision, we expected it to increase response time and constrain engagement with uncertain alternatives. Thus, we expected:

H2. Participants in the Trivia-Based Load condition will respond more slowly and will show reduced risky and exploratory choice relative to participants in the No-Trivia Control condition. Within the Trivia-Based Load condition, Higher-Load Trivia is expected to produce slower responses than Lower-Load Trivia.

Although cognitive load and anodal DLPFC stimulation were expected to exert opposing directional influences on some outcomes, two theoretical patterns were possible. Under a partially independent account, cognitive load would primarily constrain working-memory and attentional resources, whereas anodal stimulation would modulate prefrontal contributions to exploratory control, producing broadly additive effects. Alternatively, if stimulation strengthens the same processes disrupted by cognitive load, its behavioral effect could differ across Cognitive-Load Conditions, producing a compensatory or context-dependent interaction. Thus, we expected:

H3. Anodal DLPFC stimulation will increase exploratory and risk-related behavior in both the Trivia-Based Load and No-Trivia Control conditions. Under the partially independent account, the magnitude of the stimulation effect is expected to be broadly comparable across Cognitive-Load Conditions, producing an additive pattern rather than systematic moderation by cognitive load.

## 3. Methods

### 3.1. Participants

Sixty healthy adult volunteers (N = 60; age range = 18–45 years; M = 24.8, SD = 3.4; 36 males and 24 females) were recruited from science, humanities, and engineering backgrounds at the Indian Institute of Technology Mandi. Participants were randomly assigned to one of four experimental groups (n = 15 per group). All participants had normal or corrected-to-normal vision and reported no neurological, psychiatric, or major medical conditions. A safety questionnaire was used to screen for contraindications to tDCS, including a history of seizures, skin irritation at electrode sites, and use of psychoactive medication.

Written informed consent was obtained before participation. Participants were informed that they could withdraw at any time without penalty, and anonymity and data confidentiality were maintained. The study was conducted in accordance with the Declaration of Helsinki and was approved by the Institutional Ethics Committee of IIT Mandi (Approval No. IITM/IEC(H)/2025/VD/P1). Recruitment occurred between 5 April 2025 and 18 July 2025. Testing was conducted in a quiet, sound-attenuated, distraction-free room in the Applied Cognitive Science Laboratory. Participants received fixed monetary compensation independent of task performance.

### 3.2. Study design

The primary experiment followed a 2 × 2 between-subjects factorial design with Stimulation (anodal tDCS vs sham) and Cognitive-Load Condition (Trivia-Based Load vs No-Trivia Control) as between-subject factors. The four groups were anodal tDCS with Trivia-Based Load, anodal tDCS with No-Trivia Control, sham with Trivia-Based Load, and sham with No-Trivia Control. Participants in the Trivia-Based Load condition answered a trivia question before each decision trial, whereas participants in the No-Trivia Control condition proceeded directly to the decision task. Within the Trivia-Based Load condition, Trial-Level Cognitive Load was manipulated within participants by presenting 25 Lower-Load and 25 Higher-Load Trivia questions in randomized order.

Randomization was performed using a computer-generated allocation sequence before data collection. Participants were not informed of their active or sham assignment, and the sham ramp procedure was used to support participant blinding. The experimenter was aware of the stimulation condition during testing. Thus, the study followed a single-blind design. Blinding success was not formally assessed. Active or sham stimulation was completed immediately before the behavioral task.

### 3.3. Transcranial direct current stimulation

Transcranial direct current stimulation was delivered using the Neuroelectrics Starstim 32 system (Neuroelectrics, Barcelona, Spain), a wireless multichannel stimulator with real-time monitoring of electrode contact quality. Stimulation was applied through Neuroelectrics PiStim Ag/AgCl electrodes containing a 12-mm Ag/AgCl pellet (π cm² contact area) embedded in a saline-gel interface. Electrodes were positioned using a neoprene headcap according to the international 10−10 EEG system. The anodal electrode was placed at F3 to target the left DLPFC, and return electrodes were positioned over the right supraorbital region at Fp2 and AF4. The active and sham protocols are illustrated in Fig 1, and modeled voltage and electric-field distributions are shown in Fig 2.

**Fig 1:**
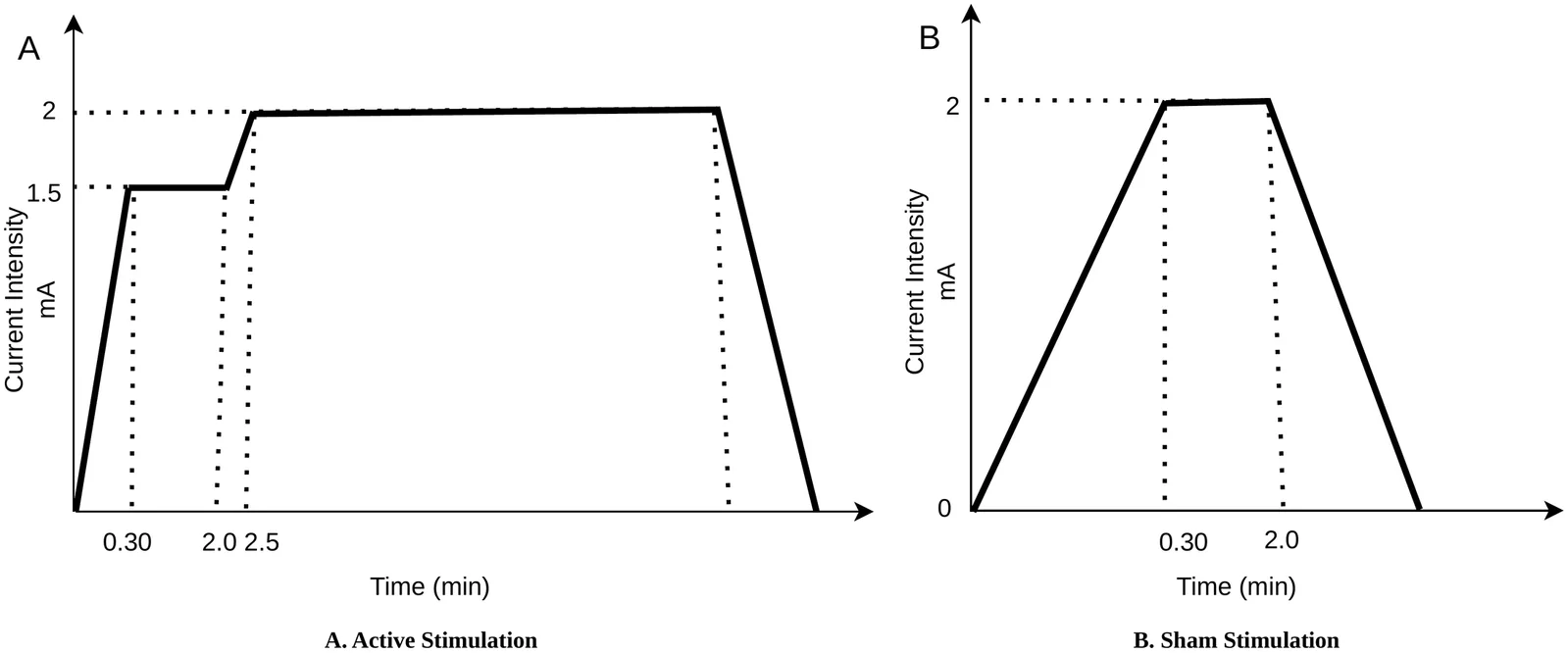
Transcranial direct current stimulation protocols. (A) Active stimulation: current was ramped to 2 mA, maintained for approximately 20 minutes, and ramped down at the end of stimulation. (B) Sham stimulation: current was ramped to 2 mA and then immediately ramped down to reproduce the initial somatosensory experience without sustained stimulation.

**Fig 2:**
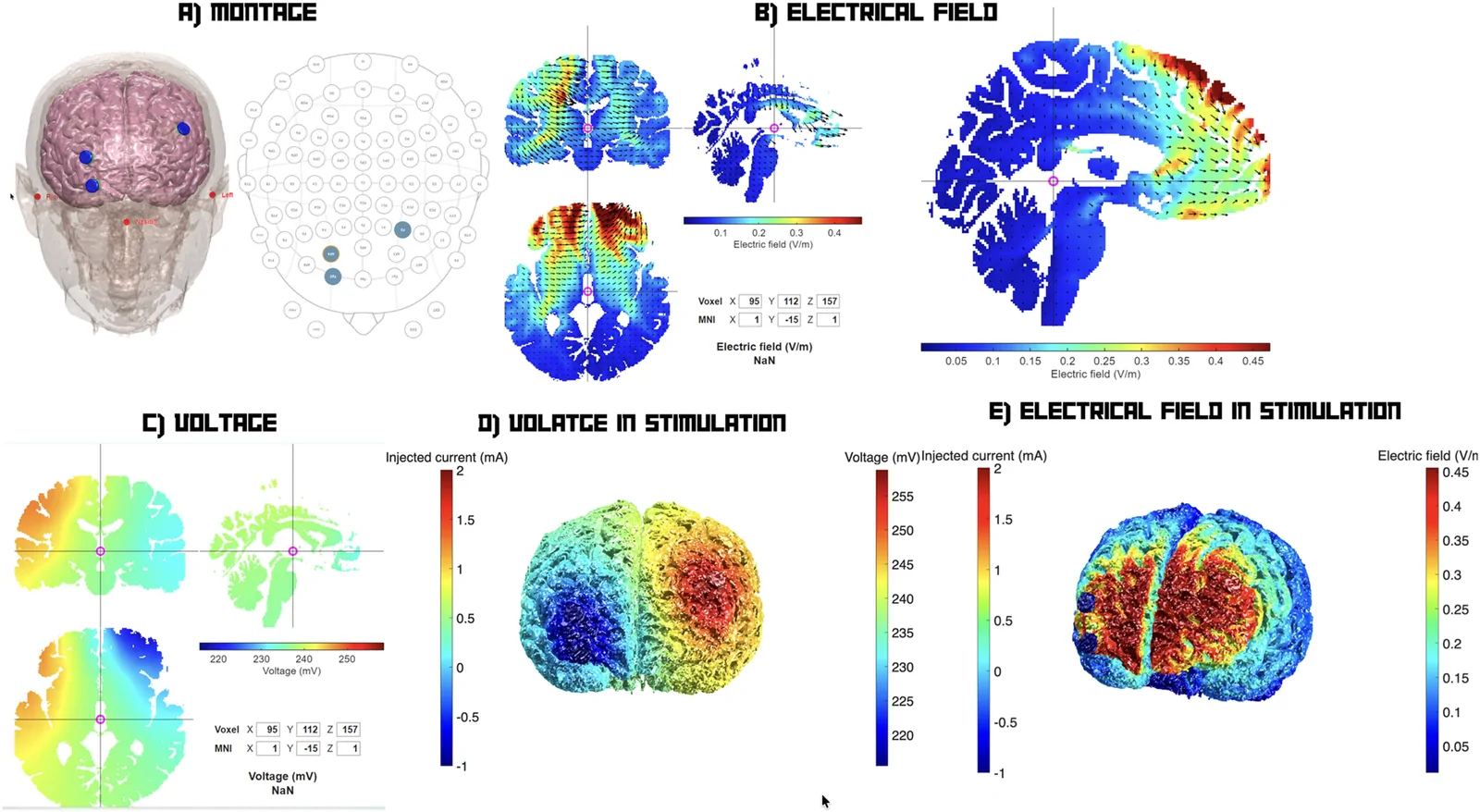
Electrode montage and modeled voltage and electric-field distributions. (A) Montage with the anode at F3 and return electrodes over the right supraorbital region at Fp2 and AF4. (B-E) Modeled electric-field magnitude and voltage distributions in cortical and orthogonal views. Simulations were computed using ROAST [29] with an applied current of 2 mA and a standard head model.

Participants in the active condition received a constant current of up to 2.0 mA for 20–22 minutes, including a 45-s ramp-up and a 30-s ramp-down. Participants were seated and asked to remain still and relaxed during stimulation. Participants in the sham condition received the same electrode setup and a 45-s ramp-up to 2.0 mA followed by immediate ramp-down, after which no sustained current was delivered. No serious adverse effects were reported; transient tingling was reported by some participants. Current-flow simulations were conducted using ROAST [29] with a standard head model and an applied current of 2 mA. The simulations were used to characterize the modeled field distribution of the montage rather than to establish individual target engagement.

### 3.4. Decision-making task

The decision-making task consisted of 50 trials, each requiring participants to select among three alternatives. In the Trivia-Based Load condition, each trial began with a trivia question and feedback on the trivia response, followed by the decision screen and outcome feedback. In the No-Trivia Control condition, the trial began directly with the decision screen. The task sequence is illustrated in Fig 3.

1. **Safe choice (Option A):** a guaranteed outcome between 2.5 and 3.5 points, with values jittered uniformly across trials.
2. **Risky choice (Option B):** a probabilistic outcome yielding either 0 points or a larger reward between 16 and 18 points. The probability of receiving the larger reward was approximately 20%, with minor jitter between 18% and 22%.
3. **Information-seeking choice (Option R):** a costly option that revealed the probability associated with the risky outcome on that trial. Selecting this option deducted one point before the participant proceeded to choose the safe or risky alternative.

**Fig 3:**
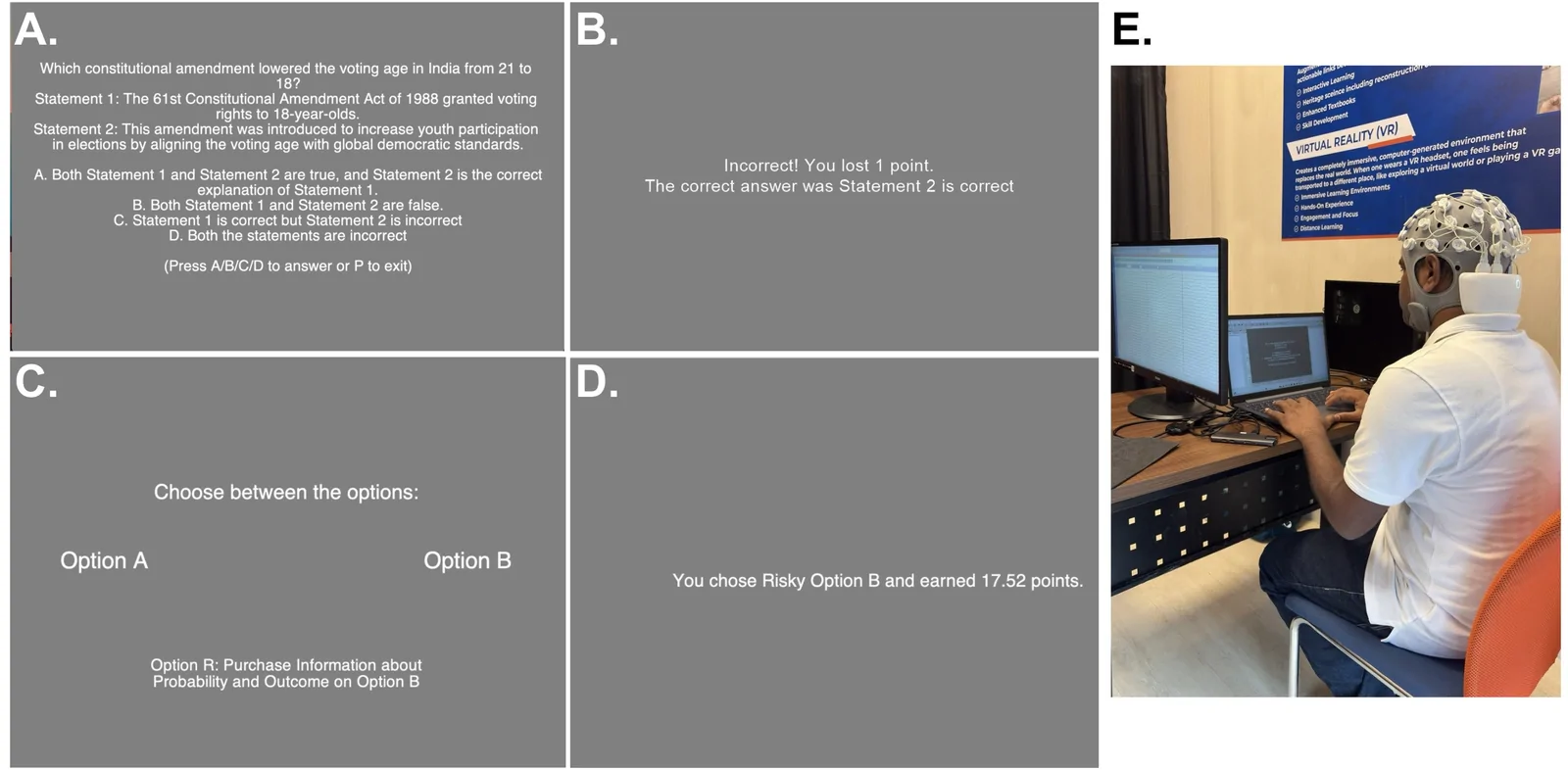
Sequence of a decision trial. (A) Trivia question presented before the decision phase in the Trivia-Based Load condition. (B) Trivia-response feedback. (C) Decision screen containing the safe, risky, and information-seeking choices. (D) Outcome feedback. (E) Example of a participant completing the task.

All stimuli were presented using PsychoPy v2023.2.3 on a 24-inch LCD monitor (1920 × 1080 pixels, 60-Hz refresh rate; BenQ, Taiwan) at an approximate viewing distance of 55 cm. The spatial positions and response keys for the safe, risky, and information-seeking choices remained fixed throughout the task to maintain a consistent response mapping. Participants responded using designated keyboard keys, and responses and reaction times were recorded automatically. A brief practice session preceded the experiment.

### 3.5. Trivia-based cognitive-load manipulation

Participants in the Trivia-Based Load condition answered one general-knowledge question before each decision trial. Questions covered science, geography, culture, mathematics, and history. Each item presented four response options, and trivia accuracy did not affect points or decision outcomes. The questions were used exclusively to impose concurrent cognitive demand.

Trial-Level Cognitive Load was manipulated through two item formats. Lower-Load Trivia consisted primarily of concise factual-recall questions with a single correct response. Higher-Load Trivia included assertion-reason items, multiple-statement items, comparative or relational judgments, and questions requiring integration of more than one information component. Each participant in the Trivia-Based Load condition completed 25 Lower-Load and 25 Higher-Load Trivia trials in randomized order. Participants in the No-Trivia Control condition did not receive trivia questions. Trivia accuracy and reaction time were recorded as indices of task engagement.

### 3.6. Behavioral outcome measures

Four dependent measures were computed. Risk-taking was defined as the proportion of trials on which the risky choice was selected. Information seeking was defined as the proportion of trials on which the costly information-seeking choice was selected. Alternation rate was defined as the proportion of successive trial transitions involving a switch between the safe choice (Option A) and the risky choice (Option B). Mean decision reaction time was calculated from the presentation of the decision screen to the participant’s response. The measures were analyzed separately because they represent related but distinct components of decision behavior.

### 3.7. Statistical analysis

#### 3.7.1. Primary between-subjects analyses.

The primary analysis consisted of a separate 2 × 2 factorial ANOVA for each dependent measure, with Stimulation (anodal tDCS vs sham) and Cognitive-Load Condition (Trivia-Based Load vs No-Trivia Control) as between-subject factors. These models tested the main effects of Stimulation and Cognitive-Load Condition and their interaction. The reaction-time analysis included all 60 participants. The combined choice-outcome dataset contained 59 complete cases; therefore, the primary factorial analyses of risky choice, information seeking, and alternation rate used N = 59. The primary analyses were interpreted outcome by outcome rather than as evidence for a single latent exploratory construct.

#### 3.7.2. Secondary trial-level mixed analyses.

The secondary analyses included the 30 participants assigned to the Trivia-Based Load condition. For each outcome, a 2 (Trial-Level Cognitive Load: Lower-Load Trivia vs Higher-Load Trivia) × 5 (Trial Block: five consecutive blocks of ten trials) × 2 (Stimulation: anodal tDCS vs sham) mixed-design repeated-measures ANOVA was conducted. Trial-Level Cognitive Load and Trial Block were within-subject factors, and Stimulation was a between-subject factor. These analyses tested whether trivia difficulty altered decision behavior within participants, whether behavior changed across the task, and whether either pattern differed by stimulation condition.

#### 3.7.3. General statistical considerations.

Analyses were performed in SPSS version 25 (IBM, Armonk, NY, USA). Statistical significance was evaluated at *p* < .05, and partial eta-squared (*ηp²)* is reported as the ANOVA effect-size measure. Homogeneity of variance was examined using Levene’s test. Sphericity was evaluated for within-subject effects with more than two levels, and Greenhouse-Geisser-corrected results were used when sphericity was violated. Exact p-values are reported except when *p* < .001.

## 4. Results

### 4.1. Primary factorial analyses: Overview

The primary factorial analyses produced different patterns across the four dependent measures. Cognitive-Load Condition significantly affected reaction time and risky choice. Stimulation significantly affected risky choice, information seeking, and alternation rate. The Stimulation × Cognitive-Load Condition interaction was significant for information seeking and alternation rate, but not for reaction time or risky choice. The findings therefore did not support a uniform additive pattern across all outcomes.

### 4.2. Reaction time

Participants in the Trivia-Based Load condition responded more slowly (M = 4.35 s, SD = 2.15) than participants in the No-Trivia Control condition (M = 3.27 s, SD = 1.99). The main effect of Cognitive-Load Condition was significant, *F*(1,56) = 4.219, *p* = .045, *ηp²* = .070. The main effect of Stimulation was not significant, *F*(1,56) = 3.114, *p* = .083, *ηp²* = .053, although mean reaction time was numerically shorter under anodal tDCS (M = 3.34 s, SD = 1.73) than under sham (M = 4.28 s, SD = 2.40). The Stimulation × Cognitive-Load Condition interaction was not significant, *F*(1,56) = 0.264, *p* = .609, *ηp²* = .005. Thus, the primary reaction-time result supported the prediction that concurrent trivia increased decision time, without evidence that this effect differed by stimulation condition (Fig 4).

**Fig 4:**
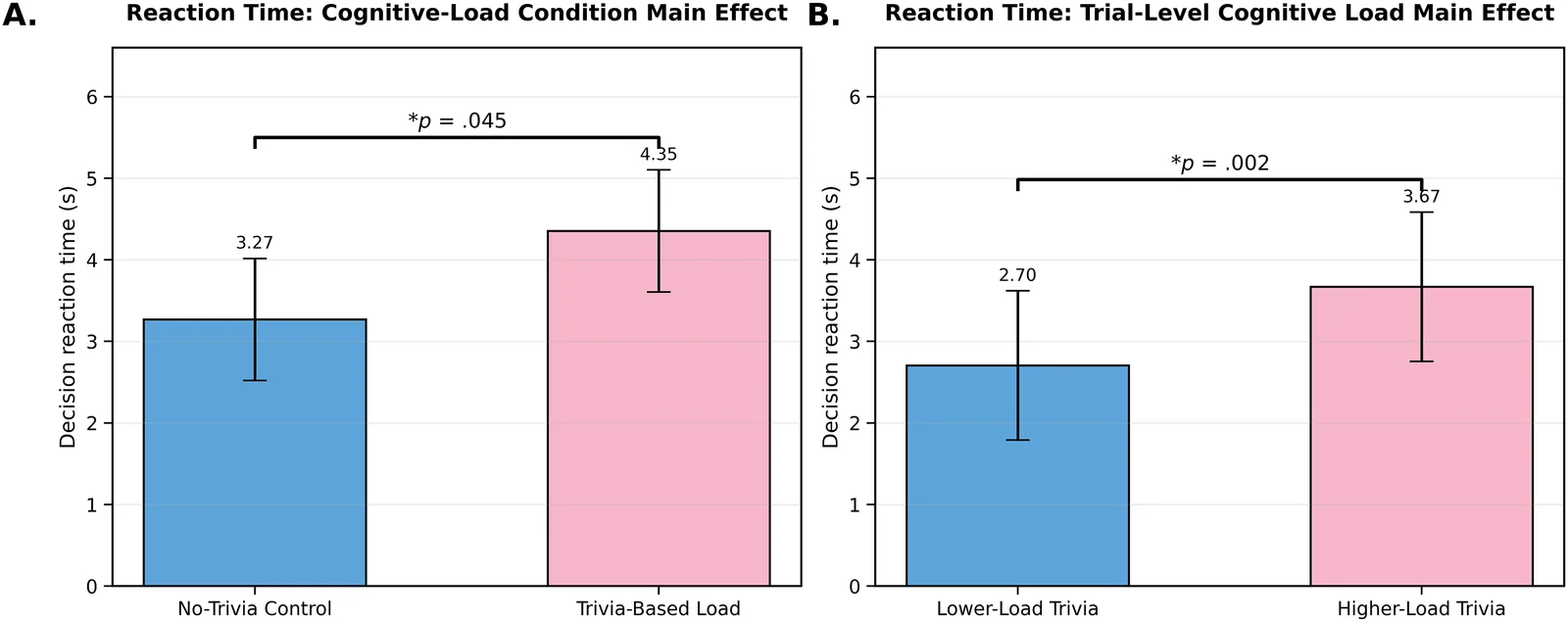
Effects of cognitive load on reaction time. (A) Mean reaction time in the No-Trivia Control and Trivia-Based Load conditions. (B) Mean reaction time for Lower-Load and Higher-Load Trivia trials in the secondary repeated-measures analysis. Error bars represent 95% confidence intervals.

### 4.3. Risky choice

Risk-taking was higher under anodal tDCS (M = .591, SD = .070) than under sham stimulation (M = .338, SD = .059), producing a significant main effect of Stimulation, *F*(1,55) = 323.285, *p* < .001, *ηp*² = .855. Risky choice was lower in the Trivia-Based Load condition (M = .428, SD = .142) than in the No-Trivia Control condition (M = .506, SD = .135), *F*(1,55) = 27.109, *p* < .001, *ηp²* = .330. The Stimulation × Cognitive-Load Condition interaction was not significant, *F*(1,55) = 0.065, *p* = .800, *ηp²* = .001. Cell means showed the same directional load-related reduction under sham (No-Trivia M = .377; Trivia-Based M = .301) and anodal tDCS (No-Trivia M = .625; Trivia-Based M = .557). This outcome supported the predicted stimulation-related increase and load-related reduction in risky choice, with an additive rather than moderated pattern (Fig 5).

**Fig 5:**
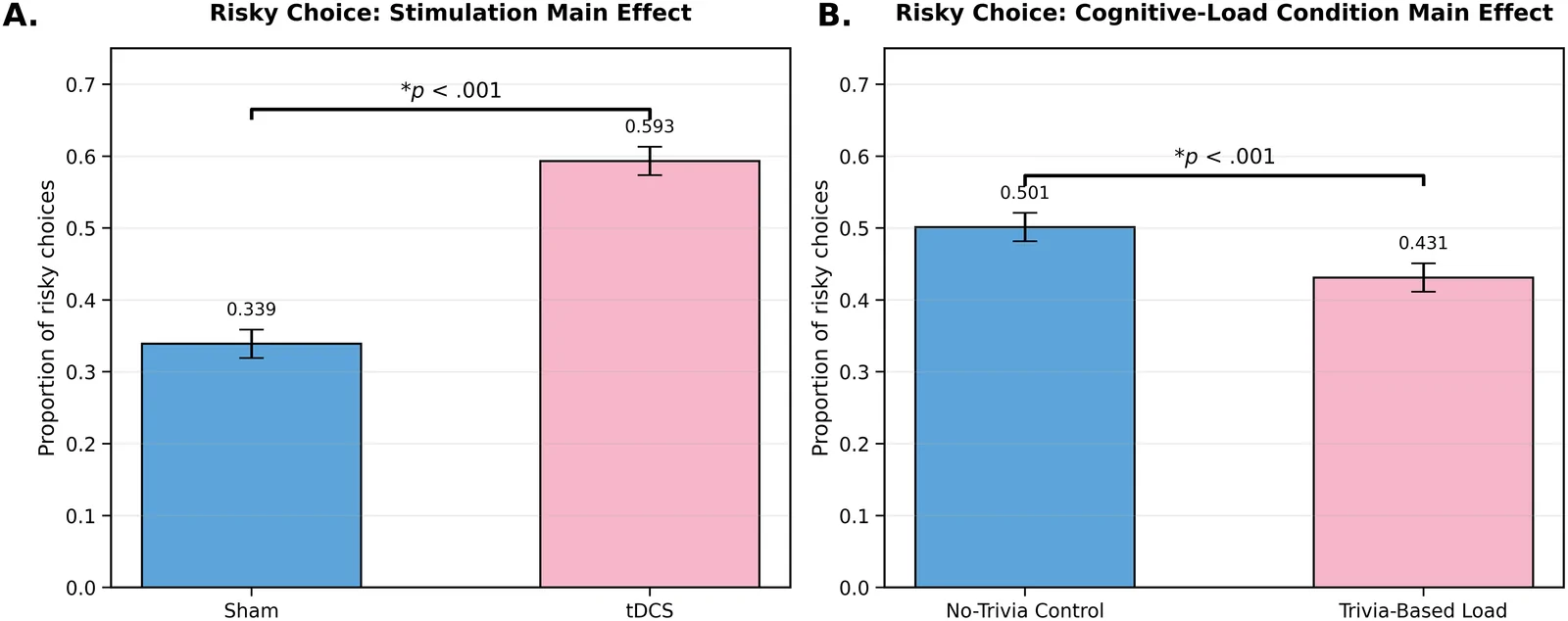
Effects of stimulation and cognitive-load condition on risky choice. (A) Mean proportion of risky choices under Sham and tDCS stimulation. (B) Mean proportion of risky choices in the No-Trivia Control and Trivia-Based Load conditions. Error bars represent 95% confidence intervals.

### 4.4. Information-seeking choice

Information seeking was higher overall under anodal tDCS (M = .313, SD = .091) than under sham stimulation (M = .261, SD = .087), *F*(1,55) = 5.916, *p* = .018, *ηp²* = .097. The main effect of Cognitive-Load Condition was not significant, *F(*1,55) = 0.010, *p* = .921, *ηp²* < .001. However, the Stimulation × Cognitive-Load Condition interaction was significant, *F*(1,55) = 14.217, *p* < .001, *ηp²* = .205. The cell means showed a crossover pattern: under sham, information seeking was lower in the Trivia-Based Load condition (M = .221) than in the No-Trivia Control condition (M = .303), whereas under anodal tDCS it was higher in the Trivia-Based Load condition (M = .352) than in the No-Trivia Control condition (M = .275). Because simple-effects tests were not included in the supplied output, the cell-specific differences are described directionally rather than interpreted as individually significant. The interaction indicates that the stimulation-related difference in information seeking depended on the concurrent cognitive-load context (Fig 6).

**Fig 6:**
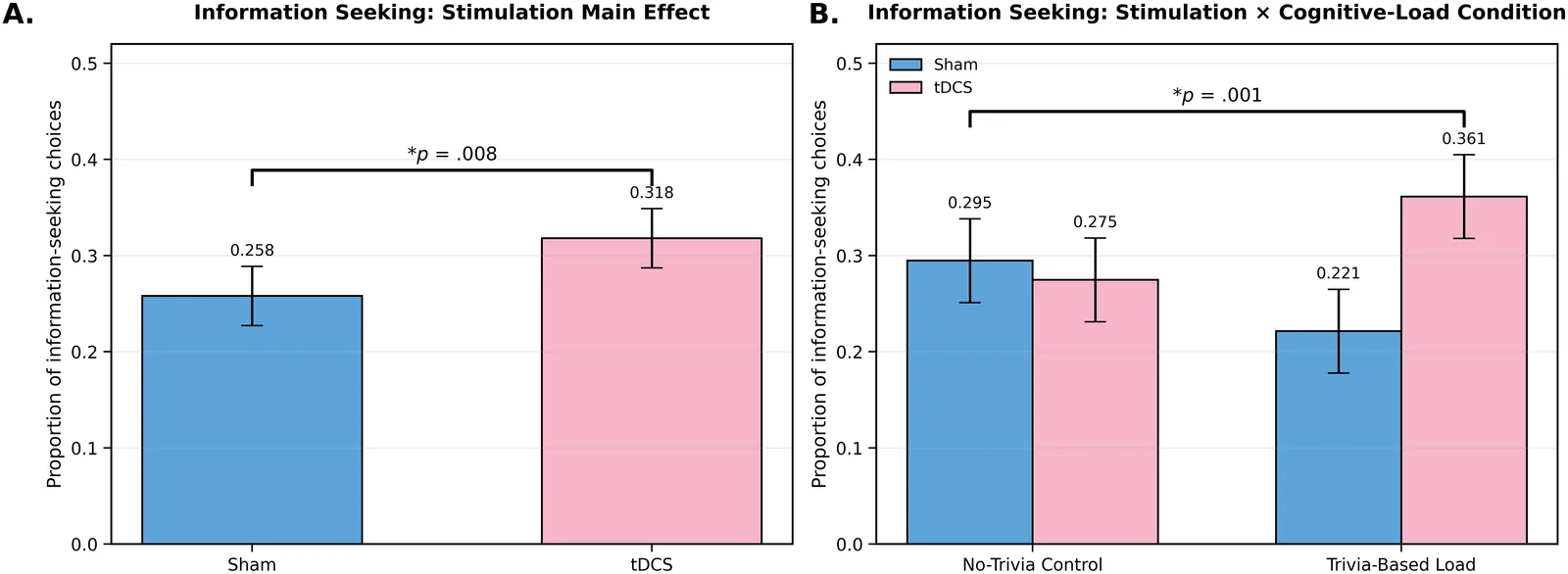
Effects of stimulation and cognitive-load condition on information-seeking choice. (A) Mean proportion of information-seeking choices under Sham and tDCS stimulation. (B) Mean proportion of information-seeking choices as a function of Stimulation and Cognitive-Load Condition. Error bars represent 95% confidence intervals.

### 4.5. Alternation rate

Alternation rate was higher overall under anodal tDCS (M = .187, SD = .081) than under sham stimulation (M = .132, SD = .047), *F*(1,55) = 10.753, *p* = .002, *ηp²* = .164. The main effect of Cognitive-Load Condition was not significant, *F*(1,55) = 1.059, *p* = .308, *ηp²* = .019. The Stimulation × Cognitive-Load Condition interaction was significant, *F*(1,55) = 6.207, *p* = .016, *ηp²* = .101. Under sham, alternation was numerically lower in the Trivia-Based Load condition (M = .120) than in the No-Trivia Control condition (M = .145), whereas under anodal tDCS alternation was numerically higher in the Trivia-Based Load condition (M = .216) than in the No-Trivia Control condition (M = .158). As with information seeking, this crossover pattern indicates context-dependent modulation rather than a uniform additive effect. Levene’s test was significant for alternation rate, *p* = .002; therefore, the magnitude of this interaction should be interpreted cautiously (Fig 7).

**Fig 7:**
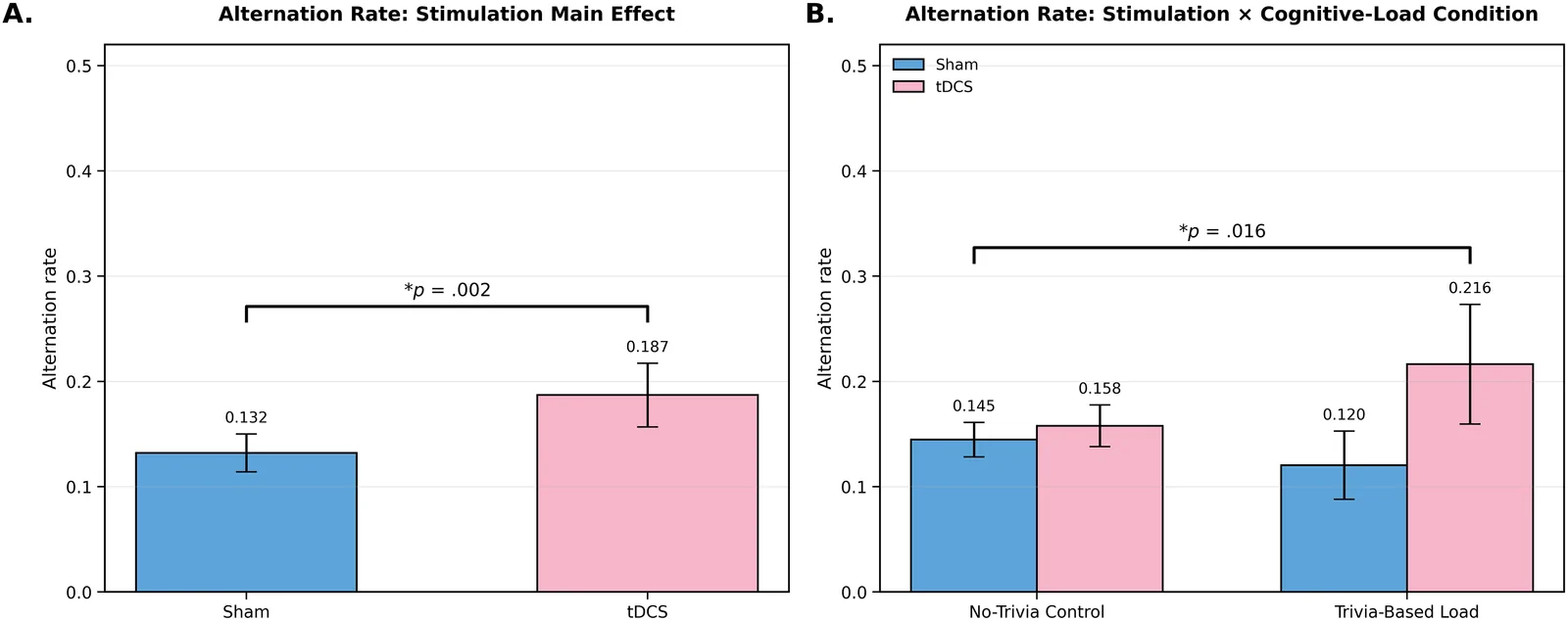
Effects of stimulation, cognitive-load condition, and trial block on alternation rate. (A) Mean alternation rate under Sham and tDCS stimulation. (B) Mean alternation rate as a function of Stimulation and Cognitive-Load Condition. (C) Mean alternation rate across the five Trial Blocks in the secondary repeated-measures analysis. Error bars represent 95% confidence intervals.

### 4.6. Secondary trial-level cognitive load and trial block analyses

Secondary mixed-design analyses were conducted on the 30 participants assigned to the Trivia-Based Load condition to examine the effects of Trial-Level Cognitive Load (Higher-Load vs. Lower-Load Trivia) and Trial Block (five consecutive blocks of ten trials) on decision-making behavior. These analyses were considered secondary because they examined differences in trivia difficulty within the Trivia-Based Load condition rather than the primary comparison between the Trivia-Based Load and No-Trivia Control conditions.

Higher-Load Trivia was associated with significantly longer decision reaction times than Lower-Load Trivia, *F*(1,28) = 11.631, *p* = .002, *ηp²* = .293, indicating that increased trivia difficulty slowed subsequent decision responses. However, the Trial-Level Cognitive Load × Stimulation interaction was not significant, *F*(1,28) = 0.062, *p* = .805, *ηp²* = .002. Likewise, no significant main effect of Trial Block or higher-order interactions involving Trial Block was observed, indicating that the effect of cognitive load on reaction time remained stable across the task. For risky choice, neither the main effect of Trial-Level Cognitive Load, *F*(1,28) = 0.002, *p* = .967, *ηp²* < .001, nor the Trial-Level Cognitive Load × Stimulation interaction, *F*(1,28) = 0.029, *p* = .865, *ηp²* = .001, was significant. The main effect of Trial Block and the remaining interactions involving Trial Block were also not significant, indicating that risky choice remained relatively stable across the five trial blocks irrespective of trivia difficulty. For information-seeking behavior, neither the main effect of Trial-Level Cognitive Load, *F*(1,28) = 0.089, *p* = .768, *ηp²* = .003, nor the Trial-Level Cognitive Load × Stimulation interaction, *F*(1,28) = 0.544, *p* = .467, *ηp²* = .019, reached significance. Likewise, neither the main effect of Trial Block, *F*(4,112) = 1.169, *p* = .328, *ηp²* = .040, nor the remaining interactions involving Trial Block was significant, indicating that information-seeking behavior remained consistent throughout the task. For alternation rate, neither the main effect of Trial-Level Cognitive Load, *F*(1,28) = 1.112, *p* = .303, *ηp²* = .038, nor the Trial-Level Cognitive Load × Stimulation interaction, *F*(1,28) = 0.500, *p* = .486, *ηp²* = .018, was significant. In contrast, a significant main effect of Trial Block was observed, *F*(4,112) = 2.591, *p* = .040, *ηp²* = .085, indicating modest variation in alternation behavior across the five trial blocks. However, no significant interactions involving Trial Block were observed, suggesting that this temporal variation was common across stimulation conditions and was not influenced by trial-level cognitive load.

Overall, these secondary analyses indicate that Trial-Level Cognitive Load primarily influenced decision reaction time, whereas risky choice, information-seeking behavior, and alternation rate were largely unaffected. Apart from modest temporal variation in alternation behavior across trial blocks, no evidence was found that Trial-Level Cognitive Load systematically modified the behavioral effects of stimulation.

## 5. Discussion

The present study examined how anodal tDCS over the left DLPFC and trivia-based cognitive demand jointly influenced experience-based decision making. The revised analysis distinguishes the primary between-subject contrast between Trivia-Based Load and No-Trivia Control from the secondary within-participant contrast between Higher-Load and Lower-Load Trivia. This distinction revealed an outcome-specific pattern. Concurrent trivia slowed decisions and reduced risky choice, while anodal tDCS was associated with greater risky choice, information seeking, and alternation. The two manipulations produced additive patterns for reaction time and risky choice, but significant interactions for information seeking and alternation. The findings therefore do not support a single conclusion that cognitive load and stimulation operated independently across all measures.

The reaction-time result indicates that performing a trivia task before each decision imposed measurable processing demands. Participants in the Trivia-Based Load condition required more time to respond than participants who proceeded directly to the decision screen. This pattern is consistent with Cognitive Load Theory and dual-process accounts, which predict that concurrent processing demands reduce the immediate availability of working-memory and attentional resources [11,12,23,25–28]. The secondary trial-level pattern, in which Higher-Load Trivia produced longer decision times than Lower-Load Trivia, further suggests that item difficulty affected processing efficiency even within the trivia condition. By contrast, the absence of reliable trial-level difficulty effects on the choice outcomes suggests that the presence of a concurrent trivia task and the difficulty of individual trivia items should not be treated as equivalent manipulations.

The secondary Trial-Level Cognitive Load analyses further clarified this distinction. Within the Trivia-Based Load condition, Higher-Load Trivia was associated with slower subsequent decision responses, whereas risky choice, information seeking, and alternation rate did not differ significantly between Higher-Load and Lower-Load Trivia trials. A modest main effect of Trial Block was observed for alternation rate, although this temporal variation did not interact with Trial-Level Cognitive Load or stimulation. These findings suggest that variation in trivia difficulty was expressed primarily in response speed rather than in the distribution of decision choices across the task.

Risky choice showed the clearest additive pattern. Trivia-Based Load reduced selection of the probabilistic high-reward alternative under both stimulation conditions, while anodal tDCS increased risky choice under both Cognitive-Load Conditions. The load-related reduction is consistent with more conservative responding when attentional resources are constrained [24]. The stimulation-related difference is consistent with previous reports that prefrontal neuromodulation can alter risk-related decisions [17,22,30]. Nevertheless, the large stimulation effect should be interpreted as a behavioral association produced by the present montage and task, not as direct evidence that a specific prefrontal computation or cortical-excitability mechanism caused the choice pattern. Information seeking and alternation produced a different result. In both outcomes, the effect of Trivia-Based Load changed direction across stimulation conditions: the trivia condition was associated with lower information seeking and alternation under sham but higher values under anodal tDCS. Information seeking required participants to pay a cost to reduce uncertainty, whereas alternation captured changes between choice types across successive trials. The parallel crossover patterns suggest that anodal stimulation altered how concurrent cognitive demand was expressed in exploratory behavior. However, information seeking may reflect strategic uncertainty reduction rather than curiosity-driven exploration alone [31,32], and alternation may reflect either flexible exploration or instability in choice preferences. The interaction for alternation should also be interpreted cautiously because homogeneity of variance was violated. These results refine the theoretical alternatives presented in the Background. Reaction time and risky choice were compatible with an additive account, because load-related differences were similar across stimulation conditions. In contrast, information seeking and alternation were compatible with a context-dependent account, because stimulation moderated the pattern associated with the Trivia-Based Load condition. A non-significant interaction for two outcomes cannot establish mechanistic independence, just as a significant interaction does not identify the neural process that generated it. The present behavioral data therefore support a mixed account in which cognitive load demands and anodal DLPFC tDCS can have both additive and interactive consequences depending on the component of decision behavior being measured.

Several limitations should be considered. First, the dependent measures are behavioral proxies rather than process-pure indicators. Risky choice, costly information seeking, and alternation capture different aspects of exploration and should not be combined into a single construct without additional modeling. Second, the positions and response keys for the three choices were fixed, which may have introduced position or key-response biases. Third, participant blinding was not formally evaluated, and the experimenter was aware of the stimulation condition. Fourth, the sample was drawn primarily from a university population, and a fixed stimulation dose was applied across individuals. Variation in individual anatomy, baseline state, and current distribution may therefore have contributed to between-participant differences [29,33,34]. Finally, cognitive load was induced through semantic trivia, and future work should test whether the pattern generalizes to visuospatial, working-memory, or multitasking demands. Future studies could integrate electroencephalography (EEG) or functional near-infrared spectroscopy (fNIRS) to examine the neural correlates underlying cognitive load and stimulation-related differences during experience-based decision making [35,36].

## 6. Conclusion

The present study combined anodal tDCS over the left DLPFC with a trivia-based cognitive-load manipulation in a repeated three-option decision-from-experience task. Concurrent trivia slowed responses and reduced risky choice, while anodal tDCS was associated with greater risky choice, information seeking, and exploratory switching. The joint pattern was not uniform: reaction time and risky choice showed no significant Stimulation × Cognitive-Load Condition interaction, whereas information seeking and alternation showed significant crossover interactions. The findings are therefore most consistent with outcome-specific modulation, including both additive and context-dependent effects. They provide behavioral evidence that cognitive demand and prefrontal stimulation jointly shape decision making under uncertainty, while leaving the underlying neurophysiological mechanisms to be established by future work.
