# Supplementary material for: Cognitive load and prefrontal neuromodulation shape exploration, risk-taking, and information seeking in experience-based decision making
Source: PLoS One. 2026 Oct 9;21(10):e0360059. doi: 10.1371/journal.pone.0360059 (PMC13652248; doi:10.1371/journal.pone.0360059)
Supplement: Supplementary file 1 [file pone.0360059.s001.docx]

Response to Reviewers

**Manuscript: Cognitive load and prefrontal neuromodulation shape exploration, risk-taking, and information seeking in experience-based decision making**

Ankit Singh, Varun Dutt, Akash K Rao

We sincerely thank the Academic Editor and both reviewers for their careful evaluation and constructive suggestions. The manuscript has been substantially revised to clarify the experimental design, distinguish the primary between-subject Cognitive-Load Condition from the secondary within-participant Trial-Level Cognitive Load manipulation, align the statistical analyses with the design, improve the theoretical framing, define the behavioral measures more precisely, and moderate causal interpretations of the tDCS findings. We have responded to each comment below and indicate the corresponding revisions in the manuscript.

# Reviewer #1

General response: We thank the reviewer for recognizing the relevance and originality of the study and for identifying the need to clarify the design, analytical hierarchy, and interpretation. The revision was organized around these three concerns. The title and abstract were softened, the Methods now separate the primary and secondary manipulations, the Results identify the primary factorial analyses before the secondary mixed analyses, and the Discussion now emphasizes outcome-specific additive and context-dependent patterns rather than a uniform claim of independence.

## Reviewer #1 - Comment Major 1

*Clarify experimental design and cognitive load manipulation. The manuscript describes the study as a 2 × 2 between-subjects design with Stimulation and Cognitive Load as between-subject factors. However, cognitive load is also described as trial-wise, within-participant, and in one section as including both low-load and high-load trivia questions, as well as a no-load condition. This creates substantial ambiguity about the actual design.
Recommendation: Please clarify whether cognitive load was manipulated between participants, within participants, or both. The abstract, Methods, Results, and Discussion should use consistent terminology. If participants were assigned to one load condition only, the manuscript should avoid describing load as a within-participant manipulation. If both load levels were presented within participants, the design and analysis should be revised accordingly.*

**Response:** We sincerely thank the reviewer for identifying this ambiguity. We revised the manuscript to distinguish two different manipulations that had previously been described using the same term. The primary between-subject factor is now termed Cognitive-Load Condition and compares Trivia-Based Load with No-Trivia Control. Within the Trivia-Based Load condition only, trivia items were classified as Lower-Load and Higher-Load Trivia; this is now termed Trial-Level Cognitive Load and is treated as a secondary within-participant factor. This distinction is used consistently throughout the Abstract, Methods, Results, Discussion, and Conclusion. We believe this revision addresses the reviewer's concern and improves the clarity and scientific rigor of the manuscript.

**Revision Made:** Abstract; Section 3.2 (Study design); Section 3.5 (Trivia-based cognitive-load manipulation); Section 3.7 (Statistical analysis); Section 4.6; and Discussion.

**Revised Text:**

“The primary experiment followed a 2 × 2 between-subjects factorial design with Stimulation (anodal tDCS vs sham) and Cognitive-Load Condition (Trivia-Based Load vs No-Trivia Control) as between-subject factors. ... Within the Trivia-Based Load condition, Trial-Level Cognitive Load was manipulated within participants by presenting 25 Lower-Load and 25 Higher-Load Trivia questions in randomized order.”

## Reviewer #1 - Comment Major 2

*Align the statistical analyses with the experimental designThe Results report analyses treating Load both as a between-subjects factor and as a within-subject factor. This makes it difficult to evaluate whether the statistical models are appropriate. It is also unclear which analysis is intended as the primary analysis, and some effects appear significant in one model but not in another. Recommendation: Please revise the statistical analysis section so that the models match the actual design. The authors should clearly identify the primary analysis for each outcome, report consistent sample sizes/degrees of freedom, and distinguish robust findings from effects that are analysis dependent. Given the repeated-trial structure and choice outcomes, the authors may also consider mixed-effects models as a more appropriate analytic approach.*

**Response:** We thank the reviewer for this important recommendation. The statistical hierarchy has been rewritten. The primary analyses are separate 2 × 2 factorial ANOVAs testing Stimulation × Cognitive-Load Condition for each outcome. The secondary analyses included the 30 participants assigned to the Trivia-Based Load condition and examined Trial-Level Cognitive Load and Trial Block as within-subject factors, with Stimulation as a between-subject factor. Sample sizes and degrees of freedom are now stated explicitly. We also distinguish findings from the primary and secondary analyses in the Results and Discussion. Because the primary outcomes were participant-level summary measures, we retained the ANOVA framework, while acknowledging trial-level mixed-effects models as an important extension for future work. We believe this revision addresses the reviewer's concern and improves the clarity and scientific rigor of the manuscript.

**Revision Made:** Section 3.7.1 (Primary between-subjects analyses), Section 3.7.2 (Secondary trial-level mixed analyses), Section 4.1 (overview), Section 4.6, Discussion.

**Revised Text:**

“The primary analysis consisted of a separate 2 × 2 factorial ANOVA for each dependent measure, with Stimulation (anodal tDCS vs sham) and Cognitive-Load Condition (Trivia-Based Load vs No-Trivia Control) as between-subject factors. ... Secondary analyses were restricted to the 30 participants assigned to the Trivia-Based Load condition. For each outcome, a 2 (Trial-Level Cognitive Load: Lower-Load Trivia vs Higher-Load Trivia) × 5 (Trial Block: five consecutive blocks of ten trials) × 2 (Stimulation: anodal tDCS vs sham) mixed-design repeated-measures ANOVA was conducted.”

## Reviewer #1 - Comment Major 3

*Clarify the theoretical logic behind the hypotheses, especially the expected absence of an interaction. The introduction presents cognitive load and anodal tDCS as opposing influences on exploration/exploitation behavior. Cognitive load is expected to reduce risk-taking and exploration, whereas anodal DLPFC stimulation is expected to increase exploratory behavior. However, the rationale for expecting additive effects rather than a Load × Stimulation interaction is not sufficiently developed. Recommendation: Please clarify why cognitive load and tDCS are hypothesized to operate through partially independent mechanisms. If tDCS could compensate for load-related disruption, then an interaction would also be theoretically plausible. The manuscript should present these alternative possibilities more clearly before stating H3.*

**Response:** We sincerely thank the reviewer. We revised the theoretical framing before H3 to present both plausible patterns rather than treating additivity as the only prediction. The revised Background now contrasts a partially independent account, which predicts broadly additive effects, with a compensatory/context-dependent account, in which stimulation could alter the behavioral consequences of cognitive demand. H3 was correspondingly rewritten to state the additive prediction more cautiously. We believe this revision addresses the reviewer's concern and improves the clarity and scientific rigor of the manuscript.

**Revision Made:** Background, paragraph immediately preceding H3 and H3.

**Revised Text:**

“Although cognitive load and anodal DLPFC stimulation were expected to exert opposing directional influences on some outcomes, two theoretical patterns were possible. Under a partially independent account, cognitive load would primarily constrain working-memory and attentional resources, whereas anodal stimulation would modulate prefrontal contributions to exploratory control, producing broadly additive effects. Alternatively, if stimulation strengthens the same processes disrupted by cognitive load, its behavioral effect could differ across Cognitive-Load Conditions, producing a compensatory or context-dependent interaction. ... H3. Anodal DLPFC stimulation will increase exploratory and risk-related behavior in both the Trivia-Based Load and No-Trivia Control conditions. Under the partially independent account, the magnitude of the stimulation effect is expected to be broadly comparable across Cognitive-Load Conditions, producing an additive pattern rather than systematic moderation by cognitive load.”

## Reviewer #1 - Comment Major 4

**Reviewer comment:** *Clarify how the behavioral measures map onto the theoretical constructs
The manuscript interprets risky choices, information-seeking choices, and alternation rates as indicators of exploration, curiosity, cognitive flexibility, or exploratory control. However, these measures may reflect partly different processes. For example, information seeking may reflect curiosity, strategic uncertainty reduction, or difficulty committing to a choice, while alternation may reflect exploration but also instability or weak value learning. Recommendation: Please define more clearly what each dependent measure is intended to capture. The authors should explain why Option B indexes risk-taking, why Option R indexes information seeking, and why alternation rate is interpreted as exploratory switching. This would strengthen the connection between the task, hypotheses, and interpretation of the findings.*

**Response:** We thank the reviewer for this helpful recommendation. We now define each behavioral measure operationally and explicitly state that the measures are related but not interchangeable. The Background explains the theoretical interpretation of risky choice, costly information seeking, and alternation, while the Methods provides formal outcome definitions. The Discussion also acknowledges alternative interpretations of information seeking and alternation. We believe this revision addresses the reviewer's concern and improves the clarity and scientific rigor of the manuscript.

**Revision Made:** Background; Section 3.6 (Behavioral outcome measures); Discussion.

**Revised Text:**

“Within the present task, selection of the probabilistic high-reward alternative was treated as an index of risk-taking because participants accepted the possibility of receiving no reward in exchange for a substantially larger potential gain than the guaranteed safe option. Selection of the costly reveal alternative was treated as an index of information seeking because participants paid one point to obtain otherwise unavailable probability information before making their final choice. Alternation rate represented exploratory switching because it measured changes between safe and risky choices across successive trials; however, higher alternation could also reflect weaker choice commitment or greater variability in choice preferences.”

## Reviewer #1 - Comment Major 5

*Soften causal claims regarding tDCS and prefrontal excitability. The manuscript uses strong causal language when describing tDCS and prefrontal excitability, including claims that the study provides a causal or mechanistic account of how prefrontal excitability shapes adaptive behavior. Although tDCS provides an experimental manipulation, its behavioral effects can vary depending on stimulation parameters, task demands, individual differences, and target specificity. Recommendation: Please soften the causal interpretation of the tDCS findings. Rather than stating that the study directly tests how prefrontal excitability shapes adaptive behavior, the authors could state that it experimentally examines whether anodal tDCS over the left DLPFC modulates decision-making behavior under cognitive load. The manuscript should also acknowledge that behavioral effects following tDCS cannot be attributed to a specific cognitive or neural mechanism without additional neural or computational evidence.*

**Response:** We sincerely thank the reviewer. We removed statements that treated the behavioral findings as direct evidence of altered cortical excitability or a specific neural mechanism. The Introduction and Background now describe tDCS as an experimental manipulation whose effects are task- and protocol-dependent. The Discussion and Conclusion explicitly state that the behavioral results do not identify a specific neural or computational mechanism, and the limitations note that the study did not include concurrent neural measurements. Future work is proposed using EEG and fNIRS to examine the neural correlates of cognitive-load and stimulation-related differences. We believe this revision addresses the reviewer's concern and improves the clarity and scientific rigor of the manuscript.

**Revision Made:** Introduction; Background; Section 3.3; Discussion; Conclusion.

**Revised Text:**

“Transcranial direct current stimulation (tDCS) provides an experimental method for examining whether modulation over the DLPFC is associated with changes in decision behavior, although the direction and magnitude of its behavioral effects depend on stimulation parameters, task demands, and individual differences. The present behavioral data therefore support a mixed account in which cognitive-load demands and anodal DLPFC tDCS can have both additive and interactive consequences depending on the component of decision behavior being measured.”

## Reviewer #1 - Comment Major 6

Figures related recommendation.
*The figures should be improved for readability and consistency. In several places, the text and numerical values appear blurry or difficult to read. I recommend increasing the figure resolution and ensuring that all axis labels, legends, numbers, and annotations are clearly visible. For figures with multiple result panels or boxes, it would also be helpful to add short titles or labels explaining what each panel/box shows. In addition, please use consistent font, font size, scaling, and subscale presentation across all figures.*

Response: We thank the reviewer for this helpful recommendation. In response, the figures have been revised for readability and consistency, with clearer panel labeling, standardized terminology, consistent fonts and scaling, and improved presentation of axis labels, legends, numerical values, and error bars. We believe these revisions address the reviewer’s concern by improving the readability, consistency, and presentation of the figures and by ensuring that the final high-resolution versions are provided as separate figure files for submission.

Revision Made: Figures 1-7 and their captions were revised for readability and consistency. Figures were standardized with consistent terminology, fonts, scaling, axis labels. The final upgraded resolution figure files will be submitted separately with the revised manuscript.

Revised Text:

“Fig 5. Effects of stimulation and cognitive-load condition on risky choice. (A) Mean proportion of risky choices under Sham and tDCS stimulation. (B) Mean proportion of risky choices in the No-Trivia Control and Trivia-Based Load conditions. Error bars represent 95% confidence intervals”.

## Reviewer #1 - Comment Minor 1

*The statements on lines 47-49 would benefit from supporting references.*

**Response:** We thank the reviewer. Supporting citations were added to the statements describing DLPFC contributions to goal maintenance, feedback integration, working memory, and attentional allocation.

**Revision Made:** Introduction and Background.

**Revised Text:**

“Concurrent cognitive load can limit working-memory and attentional resources, whereas the dorsolateral prefrontal cortex (DLPFC) supports goal maintenance, feedback integration, and flexible adjustment of behavior [2,9-13].”

## Reviewer #1 - Comment Minor 2

*Please consider adding references to support the statement on lines 50-51 and softening the causal wording, as the cognitive effects of anodal tDCS can be task- and protocol-dependent.*

**Response:** We thank the reviewer. We added supporting references and revised the text to state explicitly that tDCS effects vary with stimulation parameters, task demands, and individual differences.

**Revision Made:** Introduction and Background.

**Revised Text:**

“Transcranial direct current stimulation (tDCS) provides an experimental method for examining whether modulation over the DLPFC is associated with changes in decision behavior, although the direction and magnitude of its behavioral effects depend on stimulation parameters, task demands, and individual differences [14-21].”

## Reviewer #1 - Comment Minor 3

*“Studies that used the DLPFC” is awkward. Better: “Studies targeting the DLPFC…” (line 52)*

**Response:** We thank the reviewer. The sentence was rewritten and the broader paragraph was edited for clarity.

**Revision Made:** Background.

**Revised Text:**

“Anodal tDCS over the DLPFC has been associated with changes in working memory, risk-related choice, and exploratory behavior, but these effects are not uniform across tasks or stimulation protocols [15-21,24].”

## Reviewer #1 - Comment Minor 4

*The research questions and hypotheses are relevant, but the paragraph presenting them is somewhat difficult to follow. The wording shifts between questions and statements, and the manuscript uses future tense, such as “our research will,” even though the study has already been conducted. This reduces the clarity and professionalism of the introduction.*

**Response:** We thank the reviewer. We reorganized the Introduction and Background, removed future-tense wording, and presented the study objective and hypotheses as completed-study statements.

**Revision Made:** Introduction and Background.

**Revised Text:**

“The objective was to determine whether anodal tDCS over the left DLPFC and trivia-based cognitive demand were associated with additive or context-dependent differences in experience-based decision making.”

## Reviewer #1 - Comment Minor 5

*Please clarify the blinding procedure. The manuscript refers to a single-blind design and states that the experimenter was blind to stimulation condition, while the sham procedure is also described as maintaining participant blinding. Please specify exactly who was blinded and whether blinding success was assessed.*

**Response:** We thank the reviewer. The study used a single-blind design: participants were unaware of their active or sham assignment, whereas the experimenter knew the stimulation condition.

**Revision Made:** Section 3.2 (Study design and blinding).

**Revised Text:**

“Participants were not informed of their active or sham assignment, and the sham ramp procedure was used to support participant blinding. The experimenter was aware of the stimulation condition during testing. Thus, the study followed a single-blind design. Blinding success was not formally assessed.”

## Reviewer #1 - Comment Minor 6

*The term “stanzas” is unusual in this context. Consider replacing it with blocks, rounds, or trial blocks throughout the Results section.*

**Response:** We thank the reviewer. The term “stanzas” has been removed throughout and replaced with “Trial Block,” defined as five consecutive blocks of ten trials.

**Revision Made:** Section 3.7.2 and Section 4.6.

**Revised Text:**

“Trial Block: five consecutive blocks of ten trials.”

## Reviewer #1 - Comment Minor 7

*Please clarify whether the position of the safe, risky, and information-seeking options was fixed or counterbalanced. If positions were fixed throughout the task, the authors should briefly acknowledge the possibility of position or key-response biases.*

**Response:** We thank the reviewer. The Methods now states that option positions and response keys were fixed, and the Discussion acknowledges this as a limitation.

**Revision Made:** Section 3.4 and Discussion limitations.

**Revised Text:**

“The spatial positions and response keys for the safe, risky, and information-seeking choices remained fixed throughout the task to maintain a consistent response mapping. ... The positions and response keys for the three choices were fixed, which may have introduced position or key-response biases.”

## Reviewer #1 - Comment Minor 8

*Some wording in the Background and Discussion should be revised for clarity. For example, “must be learned feedback” should be “must be learned through feedback,” and “systemic modifications in risk-taking behavior” could be revised to “systematic changes in risk-taking behavior.”*

**Response:** We thank the reviewer. The Background and Discussion were substantially rewritten for clarity, and the awkward phrases identified by the reviewer were removed.

**Revision Made:** Background and Discussion.

**Revised Text:**
 “In experience-based decision making, individuals repeatedly sample outcomes and use feedback to decide whether to continue exploiting a familiar option or explore an uncertain alternative [1,2,4,5].”

## Reviewer #1 - Comment Minor 9

*The conclusion describes the task as a “repeated binary-choice paradigm,” but the task appears to include safe, risky, and information-seeking options. Please revise this wording so that it accurately reflects the task structure.*

**Response:** We thank the reviewer. The Conclusion now correctly describes the paradigm as a repeated three-option decision-from-experience task.

**Revision Made:** Conclusion.

**Revised Text:**

“The present study combined anodal tDCS over the left DLPFC with a trivia-based cognitive-load manipulation in a repeated three-option decision-from-experience task.”

## Reviewer #1 - Comment Minor 10

*Please avoid definitive wording such as “clearly indicates,” “robust and consistent,” or “demonstrated” when the findings are mixed across analyses. More cautious wording would better match the reported results.*

**Response:** We thank the reviewer. Definitive language was removed from the Results, Discussion, and Conclusion and replaced with more cautious, outcome-specific interpretation.

**Revision Made:** Results, Discussion, and Conclusion.

**Revised Text:**

“The findings are therefore most consistent with outcome-specific modulation, including both additive and context-dependent effects.”

## Reviewer #1 - Comment Minor 11

*The title is clear and informative, but the wording “independently shape” may overstate the conclusion. Since independence is inferred mainly from non-significant interaction effects, a more cautious title would be preferable. Maybe skip “Independently”*

**Response:** We thank the reviewer and agree. The word “Independently” was removed from the title.

**Revision Made:** Title.

**Revised Text:**

“Cognitive load and prefrontal neuromodulation shape exploration, risk-taking, and information seeking in experience-based decision making”

## Reviewer #1 - Comment Minor 12

*The abstract should describe the cognitive-load manipulation consistently with the Methods. It currently states that the study used a 2 × 2 between-subjects design but also says that cognitive load was manipulated “on a trial-wise basis.” This should be clarified to avoid confusion about whether cognitive load was manipulated between participants, within participants, or both.*

**Response:** We thank the reviewer. The Abstract now distinguishes the primary between-subject Cognitive-Load Condition from the secondary within-participant Trial-Level Cognitive Load manipulation.

**Revision Made:** Abstract.

**Revised Text:**

“The primary 2 × 2 between-subjects design crossed Stimulation (anodal tDCS vs sham) with Cognitive-Load Condition (Trivia-Based Load vs No-Trivia Control). Within the Trivia-Based Load subgroup, Lower-Load and Higher-Load Trivia were presented across trials for a secondary trial-level analysis.”

## Reviewer #1 - Comment Minor 13

*The final sentence of the abstract should be softened. Claims about “prefrontal control dynamics,” “cortical excitability,” and “independent mechanisms” go beyond what can be directly concluded from behavioral outcomes and non-significant interaction effects. A more cautious formulation would be that the findings are “consistent with additive effects of cognitive load and anodal tDCS on decision-making behavior.”*

**Response:** We thank the reviewer. The final sentence of the Abstract was rewritten to avoid mechanistic and independence claims and to reflect the observed mixture of additive and context-dependent patterns.

**Revision Made:** Abstract.

**Revised Text:**

“These findings show that cognitive load and anodal DLPFC tDCS have outcome-specific joint effects on experience-based decision making, including both additive and context-dependent patterns.”

## Reviewer #1 - Comment Minor 14

*Please consider replacing “information searching” with “information seeking” throughout the abstract and title for consistency with the rest of the manuscript.*

***Response:*** *We thank the reviewer. “Information seeking” is now used consistently throughout the manuscript.*

**Revision Made:** Title, Abstract, Methods, Results, Discussion, and figure-caption terminology.

**Revised Text:**

“Participants completed 50 decisions involving a safe choice, a probabilistic high-reward choice, and a costly information-seeking choice.”

## Reviewer #1 - Comment Minor 15

*Some reported F-values seem surprisingly large relative to the reported means and standard deviations, especially for risk-taking. For example, the risk-taking main effect of stimulation is reported as F(1,56) = 331.450 with ηp² = .855, despite group means of 0.54 vs. 0.31 and SDs of 0.14 vs 0.06. This may be correct depending on the exact model/data structure, but it should be checked carefully.*

**Response:** We thank the reviewer for prompting us to verify this result. We rechecked the participant-level risky-choice analysis, including the group coding, descriptive statistics, ANOVA output, and effect-size estimate. Because one participant had an incomplete choice record, the final risky-choice analysis used 59 complete cases. The verified stimulation effect is F(1,55) = 323.285, *p* < .001, *ηp²* = .855, as reported consistently in Section 4.3.

**Revision Made:** Section 4.3 was rechecked for consistency with the statistical output.

**Reviewer #2**

General response: We sincerely thank the reviewer for the positive assessment and for emphasizing the need to improve the organization and theoretical narrative. We substantially reorganized the Introduction, Background, Statistical Analysis, Results, and Discussion so that the rationale for each analysis and its relationship to the theoretical alternatives are explicit. We retained separate Introduction and Background headings, but shortened the Introduction and refocused the Background on the theoretical framework, operational definitions, and hypotheses to remove redundancy.

## Reviewer #2 - Comment Main 1

*Sections "Introduction" and 'Background'. It feels that these two sections contain redundant information, very summarized on the introduction, that is then re-exposed and with more elaboration in the background section. Specific hypotheses are presented only in the background section (very useful and key piece of the puzzle). I would suggest to consolidate these two sections to eliminate redundancies and make the exposition of hypothesis more concise.*

**Response:** We sincerely thank the reviewer. We agreed that the original Introduction and Background repeated the same rationale. Rather than removing the Background heading entirely, we substantially reorganized both sections: the Introduction now gives a concise overview of the problem, research gap, design, and study objective, whereas the Background is focused on the decision-from-experience framework, the behavioral constructs, the resource-limitation and neuromodulation accounts, and the hypotheses. This preserves the useful theoretical detail while minimizing repetition. We believe this revision addresses the reviewer's concern and improves the clarity and scientific rigor of the manuscript.

**Revision Made:** Introduction and Background were rewritten and shortened, with hypotheses retained in the Background.

**Revised Text:**

“The objective was to determine whether anodal tDCS over the left DLPFC and trivia-based cognitive demand were associated with additive or context-dependent differences in experience-based decision making. The following Background section develops the theoretical basis for the behavioral measures and hypotheses while avoiding repetition of the general study rationale presented above.”

## Reviewer #2 – Major comment 2

*Mixed design analysis (lin3 291). The first issue that came to my mind was that "Cognitive Load" should have been considered a within-subjects factor. After presenting analysis including Load as a between-subjects factor in the model, the authors repeated the analysis now considering (as is appropriate) Load as a within-subjects factor. I do not see why it was necessary to do the analysis with Load as between subjects factor, as the authors do not clearly explain their rationale or motivation to do so. If feels more as if they were throwing any model, they could think post hoc of at the data instead of using a model that was chosen a priori.*

**Response:** We thank the reviewer for identifying the source of confusion. The original manuscript used the same label, “Cognitive Load,” for two distinct manipulations. We now make clear that the between-subject factor is Cognitive-Load Condition (Trivia-Based Load vs No-Trivia Control), whereas the within-subject factor exists only within the Trivia-Based Load subgroup and represents Trial-Level Cognitive Load (Lower-Load vs Higher-Load Trivia). The primary 2 × 2 factorial ANOVA tests whether the presence of concurrent trivia changes behavior; the secondary 2 × 5 × 2 mixed analysis tests whether trivia difficulty and trial block produce additional within-participant variation among the 30 trivia participants.

**Revision Made:** Section 3.2; Section 3.7.1; Section 3.7.2; Section 4.1; Section 4.6; Discussion.

**Revised Text:** “These analyses were considered secondary because they examined differences in trivia difficulty within the Trivia-Based Load condition rather than the primary comparison between the Trivia-Based Load and No-Trivia Control conditions.”

## Reviewer #2 - Comment Minor 3

*In the results section, it would be much better to refer to the hypotheses explicitly, rather than writing "H1", etc. For example, lines 323-324.*

**Response:** We thank the reviewer. The revised Results no longer relies on shorthand statements such as “H1 supported” or “H2 supported.” Instead, each subsection states the substantive prediction and describes whether the observed outcome was consistent with it.

**Revision Made:** Results Sections 4.2-4.5 and Discussion.

**Revised Text:**

“This outcome supported the predicted stimulation-related increase and load-related reduction in risky choice, with an additive rather than moderated pattern.”

## Reviewer #2 - Comment Minor 4

*This is also the case for references to "Option B", "Option R", etc. Readability would greatly improve if these instances read explicitly as "Risky Choice" or "Information-seeking choice" in the text and figures.*

**Response:** We thank the reviewer. We now use “Risky choice” and “Information-seeking choice” throughout the Results, Discussion, table, and figure-caption terminology. The option labels are retained only in the Methods where the task alternatives are formally defined.

**Revision Made:** Section 3.4; Sections 4.3-4.4; Discussion; figure-caption terminology.

**Revised Text:** “Risk-taking was higher under anodal tDCS ... than under sham stimulation .... ... Information seeking was higher overall under anodal tDCS ... than under sham stimulation.”
